# Applicability of DNA pools on 500 K SNP microarrays for cost-effective initial screens in genomewide association studies

**DOI:** 10.1186/1471-2164-8-214

**Published:** 2007-07-04

**Authors:** Sophia J Docherty, Lee M Butcher, Leonard C Schalkwyk, Robert Plomin

**Affiliations:** 1 Social, Genetic and Developmental Psychiatry Centre, Box Number P082, Institute of Psychiatry, DeCrispigny Park, London, SE5 8AF, UK

## Abstract

**Background:**

Genetic influences underpinning complex traits are thought to involve multiple quantitative trait loci (QTLs) of small effect size. Detection of such QTL associations requires systematic screening of large numbers of DNA markers within large sample populations. Using pooled DNA on SNP microarrays to screen for allelic frequency differences between groups such as cases and controls (called SNP Microarray and Pooling, or SNP-MaP) has been validated as an efficient solution on both 10 k and 100 k platforms. We demonstrate that this approach can be effectively applied to the truly genomewide Affymetrix GeneChip^® ^Mapping 500 K Array.

**Results:**

In comparisons between five independent DNA pools (*N *~200 per pool) on separate Affymetrix GeneChip^® ^Mapping 500 K Array sets, we show that, for SNPs with minor allele frequencies > 0.05, the reliability of the rank order of estimated allele frequencies, assessed as the average correlation between allele frequency estimates across the DNA pools, was 0.948 (average mean difference across the five pools = 0.069). Similarly, validity of the SNP-MaP approach was demonstrated by a rank-order correlation of 0.937 (average mean difference = 0.095) between the average DNA pool allele frequency estimates and the allele frequencies of an independent (CEPH) sample of 60 unrelated individually genotyped subjects.

**Conclusion:**

We conclude that SNP-MaP can be extended for use on the Affymetrix GeneChip^® ^Mapping 500 K Array, providing a cost-effective, reliable and valid initial screen of 500 K SNP microarrays in genomewide association scans.

## Background

The post-genomic era signals increased confidence in the possibility of locating quantitative trait loci (QTLs) that underpin the heritability of common complex disorders. However, problems still remain and progress towards reliably detecting QTLs for complex disorders, for which multiple genetic and environmental risk factors are responsible, has been slower than expected [1]. Hypothesis-driven candidate gene studies are important, but with approximately 25,000 genes in the human genome it is often difficult to predict how variation in gene product will affect a particular phenotype. Moreover, it may be a mistake to limit the search for QTLs to the 2% of the genome that codes for proteins, rather than using a genomewide strategy that considers non-coding as well as coding DNA sequences [2]. Linkage designs represent a genomewide approach but are limited to detecting QTLs of relatively large effect size [3,4]. Association designs are needed to provide genomewide searches for QTLs of small effect size but hundreds of thousands of DNA markers genotyped on samples of thousands of individuals are needed to detect QTLs of small effect size [5].

Microarrays that permit highly multiplexed genotyping greatly reduce this genotyping burden. Several companies have developed microarrays to meet the need for genomewide association analysis (most notably Affymetrix™ and Illumina™). In each case, single nucleotide polymorphisms (SNPs) are the marker of choice because they are bi-allelic, abundant throughout the genome and relatively stable from generation to generation [6]. Alleles of SNPs close together on a chromosome will be correlated (that is, in linkage disequilibrium) and thus they are also likely to be associated with a QTL in between them, known as indirect association [7,8].

The number of SNPs required for an indirect genomewide association study depends on several factors, including recombination frequencies, effect size, and sample size[9]. It is estimated however, that approximately 500,000 'randomly chosen' SNPs or approximately 250,000 well-chosen 'tag SNPs', which take into account patterns of linkage disequilibrium, are adequate to capture nearly all common variation in Caucasian, Han Chinese, and Japanese populations [10,11].

With the advent of microarrays that genotype hundreds of thousands of SNPs, genomewide association studies are becoming a reality. For example, microarrays have been instrumental in genomewide association scans that discovered an intronic SNP in complement factor H (CFH) causing age related macular degeneration [12], and a non synonymous SNP in *IL23R *– a gene encoding a subunit of a proinflammatory cytokine interleukin-23 receptor – that confers susceptibility to Crohn's disease [13]. However, these associations involve large effect sizes with odds ratios greater than 3.0; very large samples will be needed to detect smaller QTL effects.

The solution appears simple: Use microarrays to genotype large cohorts for hundreds of thousands of SNPs. However, despite the high throughput of SNP genotyping microarrays and the low cost per genotype, the cost of individually genotyping a sample of even 1000 individuals remains prohibitive outside of large-scale consortia. Until genotyping becomes even cheaper, one solution is to screen the genome using DNA pools on microarrays to nominate SNPs. DNA can be pooled for large samples of cases and controls or the low and high extremes of a quantitative trait and the pooled DNA can be genotyped on SNP microarrays, a method we call SNPMicroarrays and DNA Pooling (SNP-MaP). The technique of DNA pooling has been validated using both microsatellites [e.g., [14-17]] and SNPs [e.g., [18-25]] on several genotyping platforms, including the Affymetrix 10 K microarray [26-32], 100 K microarray [33], and one half of the two-chip 500 K microarray set [34]. The main advantage of DNA pooling is that it provides average allele frequency estimates for a group rather than genotyping each individual in the group and then averaging their allele frequencies statistically. The main limitation is that individual genotypes and haplotypes cannot be extracted because the DNA of individuals is pooled.

The confirmation of previously identified associations with rheumatoid arthritis have demonstrated the feasibility of SNP-MaP case-control study designs for detecting susceptibility alleles to complex diseases [35]. Furthermore, substantive studies have already used pooled DNA across a variety of microarray platforms to detect novel SNP associations. SNP-MaP has been used as an initial screen in the identification of four susceptibility loci for mild mental impairment [36], 11 SNPs associated with reading ability [37], and several SNPs, including an intronic SNP from the diacylglycerol kinase eta (DGKH) gene, associated with bipolar disorder [38]. These studies were performed using Affymetrix 10 K, Affymetrix 100 K and Illumina HumanHap550 microarrays, respectively. Moreover, the Affymetrix GeneChip^® ^Mapping 500 K Array set has already been used to allelotype DNA pools in substantive research, implicating a *KIBRA*-encoding locus in memory performance [39].

In this report we evaluate the applicability of DNA pools on the first truly genomewide, commercially available genotyping platform, the Affymetrix 500 K GeneChip^®^, which affords significantly greater coverage of all common variation than do 10 K and 100 K arrays [10]. Although already employed in substantive research, three subtle but potentially detrimental changes differentiating the Affymetrix 500 K GeneChip^® ^from its validated 10 K and 100 K predecessors, deem validation of the full 500 K set with pooled DNA desirable. These changes are: 1) the introduction of two new restriction digest endonucleases, NspI and StyI; 2) a decrease in feature size from 8 μm to 5 μm; and 3) a reduction from 40 to 24 probes per SNP for 90% of the 500 K microarray.

To ascertain the reliability and validity of genomewide screening using DNA pools, we assayed five previously validated, independent DNA pools (*N *~200 independent individuals per pool) separately on Affymetrix GeneChip^® ^Mapping 500 K Array sets. To assess reliability, the allele frequency estimates were compared across the five DNA pools. To assess validity, the average allele frequency estimates across the five pools were compared with a CEPH sample of 60 individuals from the HapMap project [11] previously genotyped using the Affymetrix 500 K GeneChip^®^.

## Results

### Detection rates

All five DNA pools produced similar detection rates with the 500 K GeneChip^®^; rates varied from 87.9 to 97.5% for the Sty array and 92.3 to 97.9% for the Nsp array. These detection rates for the 500 K GeneChip^® ^were similar to those from our previous work using pooled DNA on the 10 K and 100 K GeneChip^® ^platforms [29,32,33] and only slightly less than for individual genotyping of the reference DNA sample provided by Affymetrix (99.3% for Sty, 98.9% for Nsp).

### Allele frequency estimation

Rather than deriving separate RAS scores for sense and anti-sense quartets, allele frequencies can be estimated more reliably using a composite measure. Thus, allele frequency estimates were calculated using a modified form of the RAS score algorithm (RAS_av-all_) based upon an average of all quartet measures.

### Reliability

Reliability was assessed by correlating allele frequency estimates across the five DNA pools using the Pearson correlation coefficient (*r*), as well as calculating their average absolute differences. As can be seen from Table 1, estimates of allele frequency across the 500 K microarray are highly reliable (*N *= 457,607 – 487,666 SNPs for which 70% of quartet measurements were available). The average correlation among the five DNA pools was 0.956 and their average absolute difference was 0.066.

**Table 1:** Reliability of allele frequency estimates in DNA pools for SNPs on the Affymetrix 500 K microarray.

Array	All SNPs			MAF > .05		
	Worst	Best	Average	Worst	Best	Average
NspI (250 k)	0.952 (0.069)	0.969 (0.056)	0.957 (0.063)	0.934 (0.072)	0.963 (0.058)	0.947 (0.065)
StyI (250 k)	0.944 (0.075)	0.968 (0.067)	0.955 (0.070)	0.944 (0.079)	0.964 (0.070)	0.949 (0.073)
Both (500 K)	0.949 (0.071)	0.962 (0.062)	0.956 (0.066)	0.94 (0.075)	0.956 (0.065)	0.948 (0.069)

Values are mean Pearson correlation coefficients (r) between the 5 DNA pools using the average of all quartet estimates (RAS_av-all_). 'Worst' and 'Best' refer to the 10 bivariate comparisons between the 5 DNA pools and 'Average' is the average of these 10 correlations. Values in parentheses are mean absolute differences between DNA pools. 'All SNPs' includes all SNPs on the array (including rare and non-polymorphic SNPs) for which 70% of quartet measurements were available (N = 457,607 – 487,666 across both microarrays). 'MAF > .05' only includes SNPs with minor allele frequency greater than .05 (N = 428,179 – 456,241 across both microarrays).

It is possible that these estimates of reliability are inflated by the inclusion of low frequency alleles; particularly in the case of non-polymorphic alleles when all SNPs are considered. To control for this we re-ran these analyses using only SNPs with MAF > .05 (*N *= 428,179 – 456,241 SNPs). Table 1 indicates similar results for correlations (0.948) and mean differences (0.069).

Because we employ multiple biological replicates (constructed singly) without technical replicates it is difficult to decompose variance attributable to microarray measurement and pooling construction. However, a recent paper using one microarray of the two-microarray Affymetrix 100 K set has estimated that the microarray component of variance is up to seven times greater than that of pool construction (variance due to microarray ≈ .00126 *vs. *variance due to pool construction ≈ .00018; [see [40]]). Given the relationship between 500 K and 100 K performance (see below), we would expect to see similar estimates of microarray variance for the 500 K microarray.

### Validity

To assess validity, we compared our estimates of allele frequencies from pooled DNA with individual genotyping data from an independent sample of 60 CEPH individuals. All the CEPH individuals (as well as Han Chinese, Japanese and Yoruban populations) have been genotyped for SNPs on the Affymetrix 500 K microarray; these data, which have been acquired using multiple genotyping platforms, are available for download from the HapMap project [11]. Considering the small CEPH sample size, Table 2 indicates that the SNP-MaP approach exhibited reasonable validity, reflected by high correlations (0.926 on average for both arrays) and modest mean differences (0.100) between each pool and the CEPH population, with similar results for MAF > .05. We found no difference between array-type (Nspl or Styl) on indices of reliability or validity, regardless of whether all SNPs or just common SNPs (MAF > .05) were included.

**Table 2:** Validity of allele frequency estimates in DNA pools for SNPs on the Affymetrix 500 K microarray.

Array	All SNPs			MAF > .05		
	
	Worst	Best	Average	Worst	Best	Average
NspI (250 k)	0.913 (0.108)	0.942 (0.089)	0.928 (0.099)	0.902 (0.113)	0.935 (0.093)	0.918 (0.103)
StyI (250 k)	0.903 (0.114)	0.943 (0.089)	0.925 (0.101)	0.889 (0.119)	0.936 (0.093)	0.914 (0.106)
Both (500 K)	0.909 (0.111)	0.935 (0.094)	0.926 (0.100)	0.896 (0.116)	0.927 (0.098)	0.916 (0.104)

Values are average Pearson correlation coefficients (r) between the allele frequency estimates DNA pool and an independent population (CEPH) of 60 individuals. 'Worst' and 'Best' refer to the 10 bivariate comparisons between the 5 DNA pools and 'Average' is the average of these 10 correlations. Values in parentheses are mean absolute differences. 'All SNPs' includes all SNPs on the array including rare and non-polymorphic SNPs (N = 470,512 – 487,666). 'MAF > .05' only includes SNPs with minor allele frequency greater than .05 (N = 440,401 – 459,418).

As expected, validity was further improved when all five DNA sub-pool estimates were aggregated, supporting the use of multiple DNA sub-pools in SNP-MaP studies [33]. After excluding SNPs whose average minor allele frequency across the five pools was less than .05 or which had fewer than four pools, SNP-MaP estimates correlated 0.937 (mean difference = 0.095) with the CEPH population (*N *= 412,626 SNPs). Standard errors of the mean (SEM) across at least four replicates were small (approximately 60% of the data exhibited SEMs < 0.025 and 90% of the data exhibited SEMs < 0.045) but were predictive of validity, with smaller variance across replicates indicating greater accuracy. We estimate that with at least 4–5 biological replicate DNA pools, the SNP-MaP method has 80% power to detect allele frequency differences between case and controls on the order of .043 for rarer alleles (.05 < MAF < .10) and .095 for common alleles (.45 < MAF < .5).

### Artificial pooling experiment as an indication of reliability

After the removal of rare alleles (MAF < .05), the average correlation between the allele frequencies of two simulated biological replicate DNA pools – each containing the individual genotypes of 30 unrelated individually genotyped CEPH individuals – was calculated as 0.959. This provides an expected correlation between two pools containing independent samples and no technical variance (microarray or pool construction). Although the difference between 0.959 and 0.948 (our observed estimates of reliability) is small, with a p-value of 0.01 it is significantly different, however; this significance should be interpreted with caution as this is likely to reflect the sheer immensity of SNPs correlated and does not act as a summary statistic that can be used to quantify case-control allele frequency differences across the microarray.

### Reliability and validity of the 500 K GeneChip^® ^versus 100 K GeneChip^®^

Because the Affymetrix 500 K GeneChip^® ^Mapping Array shares 27,281 probe-sets with the 100 K GeneChip^®^, we investigated how similar the two platforms performed with the same DNA pools on the same SNPs. Overall, the 500 K platform performed slightly less well than the 100 K platform both in terms of reliability and validity. Using RAS_av-all_, indices of reliability (between DNA pool comparisons) for the 100 K microarray ranged from .958 to .977 (mean = .967), whereas for the 500 K these correlations ranged from .933 to .949 (mean .940). This trend was mirrored in terms of validity when comparisons with the CEPH population were considered (data not shown).

## Discussion

The ability to screen common SNPs for allele frequency differences with DNA pools is now feasible on a genomewide scale using the Affymetrix 500 K GeneChip^® ^Mapping Array. We allelotyped five previously validated DNA pools on the 500 K microarray and show high reliability and validity for more than 500,000 SNPs.

It is important to note that estimates of reliability and validity, although high, were lower than those obtained using the 10 K [36] and 100 K [33] platforms . Comparing our previously published results for the 100 K platform versus the present results for the 500 K platform, the average reliability correlation was 0.969 for the 100 K vs. 0.948 for the 100 K (average absolute difference: 0.054 vs. 0.069); the average validity correlation was 0.939 vs. 0.916 (average absolute difference: 0.081 vs. 0.104). A reduction in both feature size and in the number of features per SNP may be accountable for this decline in performance from the 100 K array to the 500 K array. Nonetheless, the performance of the 500 K array is adequate, especially in comparison to the basic sampling variation seen in our artificial pools using the individually genotyped CEPH sample where no measurement error was present. Concerns may arise with Affymetrix's latest release, the SNP array 5.0, which sees all SNPs from the two-chip 500 K set, along with 420,000 additional non-polymorphic probes which may be used to assess copy number variation, contained within a single microarray. As our analysis currently employs mis-match probe data, it remains to be seen if the reduction in the number of features per SNP required for such multiplexing will further reduce the reliability and validity of pooled DNA allele frequency estimates.

Thus, we conclude that allele frequency estimates from DNA pools appear reliable on the 500 K platform as well as 100 K and 10 K platforms to screen for allele frequency differences between groups. Despite the reliability and validity found for pooled DNA, three limitations of this study should be mentioned, which put these results in an even more favourable light. Firstly, in terms of validity, these data are uncorrected for differential hybridisation kinetics, which can result in unequal representations of SNP alleles [27,31,33,41,42]. This is unimportant for individual genotyping as allele-calling algorithms routinely process – and, in the case of homozygotes, actually benefit from – discordant allele fluorescence values. If DNA pooling is used to estimate absolute allele frequencies, certain estimates will be biased when unequal allelic representation occurs. However, DNA pooling is rarely used to estimate absolute allelic frequencies. DNA pooling is usually used to assess relative differences between groups such as cases and control; previous reports of differential hybridisation [27] indicating that the proportion of SNPs exhibiting differential hybridisation (likely to result in type I and type II errors) is small, suggests all pools are subject to similar technical variation and thus allelic bias. A suitable next step however, is to identify the specific SNPs that exhibit large differential hybridisation and either omit these from subsequent analysis or correct in the appropriate manner [e.g., k correction; see [42,43]].

Secondly, the CEPH population that we used to determine validity is a relatively small sample, which will have undoubtedly reduced estimates of validity. Thirdly, reliability was assessed as the average difference between just one pool and another, rather than the difference between groups using multiple sub-pools for each group. It is therefore likely that our estimates of reliability are conservative underestimates, as may be inferred by the distribution of small SEMs.

DNA pooling involves several limitations. With DNA pooling it is not possible to extract individual genotypic information to allow analyses of individual differences or haplotypes. In addition, once individuals have been pooled they cannot be 'unpooled', thus tethering tests of allele frequency differences to the phenotype used for pooling [44]. However, these issues are offset by the considerable financial benefits of DNA pooling.

As with individual genotyping, issues of multiple testing and false positive results are critical for genomewide association analyses using SNP microarray. As one might expect, replication has been demonstrated to improve estimates of allele frequency, and therefore may be used to reduce, although by no means eliminate, the dilemma posed by false positive results [45]. Although no consensus has yet been reached as to the fairest method of analysing the enormous volume of data generated by genome-wide studies, especially for identifying QTLs of small effect size, progress with regard to high-throughput microarrays is being made [46]. Regardless of which statistical procedures are agreed upon that demonstrate adequate association, the ultimate criterion for association must be independent replication. The expense of individually genotyping large samples using SNP arrays makes replication of genomewide association scans unlikely. However, because DNA pooling is relatively inexpensive, SNP-MaP strategies will facilitate replication on a genomewide scale.

## Conclusion

With results for reliability and validity similar to those previously demonstrated on 10 k and 100 k arrays, we have shown that the SNP-MaP approach can be applied to a 500 k platform. We conclude that the Affymetrix 500 K GeneChip^® ^Mapping Array can be used in SNP-MaP studies to provide an efficient, reliable, and valid genomewide screen of allele frequency differences between groups, thus facilitating the detection of SNPs of small effect size.

## Methods

### Samples

Five independent pools of DNA were created from a sample of 1028 white Caucasian individuals (538 females and 490 males) randomly selected from a representative community-based sample of more than 14,000 children in the Twins Early Development Study, which we used in a SNP-MaP study of cognitive ability and disability with the 10 K microarray [36].

### DNA quantification and pool construction

DNA samples were extracted from buccal swabs [47], quantified using a spectrophotometer (260 nm) and diluted to a target concentration of 50 ng/μl. Each sample was subsequently quantified in triplicate using fluorimetry (employing PicoGreen^® ^dsDNA quantitation reagent, Cambridge Bioscience, UK) and samples that were accurately quantified (± 0.5 ng/μl) were accepted for pooling. Each individual's DNA was randomly assigned to one of five DNA pools, thus providing five independent pools with 204–206 individuals. Each individual contributed 79.1 ng of DNA to a DNA pool. Each pool concentration ranged from 13.33 to 13.57 ng/μl.

### SNP microarray allelotyping of pooled DNA

Because pooled DNA can be used only to estimate allelic frequency, not genotypic frequency, we refer to allelotyping rather than genotyping. Each of the five DNA pools was allelotyped using the GeneChip^® ^Mapping 500 K Array set in accordance with the standard protocol for individual DNA samples (see the GeneChip^® ^Mapping 500 K Assay Manual for full protocol). Each microarray was scanned using the GeneChip^® ^Scanner 3000 with High-Resolution Scanning Upgrade, which was controlled using GeneChip^® ^Operating software (GCOS) v1.4. Cell intensity (.cel) files were analyzed using GTYPE. Each of the five DNA pools was assayed on a separate microarray set; for quality control checks, a reference DNA individual provided by the manufacturer (sample number 100103) was also assayed on a separate microarray set.

### Generation of SNP-MaP allele frequency estimates

Relative Allele Signal (RAS) scores, calculated using the 10 K MPAM Mapping algorithm, have been shown to be reliable and valid indices of allele frequency in pooled DNA [26-32]. Provided by Affymetrix, the GTYPE user's manual contains a full description of the Affymetrix Mapping GeneChip^® ^probe-sets and how they are used to calculate RAS scores. Briefly, a RAS score for each SNP is derived from multiple 'quartet' measures. Quartets contain four 25 bp sequences (probes) with variations on the central base. The central base of the probe-set corresponds to two perfect match (*PM*) probes and two mismatch (*MM*) probes for each allele of the SNP, allele A (*PM*_A _and *MM*_A_) and allele B (*PM*_B _and *MM*_B_). There is a 90:10 split between 6 and 10 quartet measures per SNP on the 500 K microarray set. These quartets occur either exclusively on the sense or anti-sense strand, or on both strands. The distribution of quartets relative to the SNP site varies from SNP to SNP but can include up to seven quartets up or downstream of the SNP site ('off-sets') including the SNP site itself ('zero offset') on a single strand. After subtracting the average mismatch intensity (MM¯
 MathType@MTEF@5@5@+=feaafiart1ev1aaatCvAUfKttLearuWrP9MDH5MBPbIqV92AaeXatLxBI9gBaebbnrfifHhDYfgasaacH8akY=wiFfYdH8Gipec8Eeeu0xXdbba9frFj0=OqFfea0dXdd9vqai=hGuQ8kuc9pgc9s8qqaq=dirpe0xb9q8qiLsFr0=vr0=vr0dc8meaabaqaciaacaGaaeqabaqabeGadaaakeaadaqdaaqaaiabd2eanjabd2eanbaaaaa@2F03@) from each *PM *probe, a RAS score for each quartet is generated by calculating the ratio of A to A+B fluorescence values. If MM¯
 MathType@MTEF@5@5@+=feaafiart1ev1aaatCvAUfKttLearuWrP9MDH5MBPbIqV92AaeXatLxBI9gBaebbnrfifHhDYfgasaacH8akY=wiFfYdH8Gipec8Eeeu0xXdbba9frFj0=OqFfea0dXdd9vqai=hGuQ8kuc9pgc9s8qqaq=dirpe0xb9q8qiLsFr0=vr0=vr0dc8meaabaqaciaacaGaaeqabaqabeGadaaakeaadaqdaaqaaiabd2eanjabd2eanbaaaaa@2F03@ > *PM*_A _OR MM¯
 MathType@MTEF@5@5@+=feaafiart1ev1aaatCvAUfKttLearuWrP9MDH5MBPbIqV92AaeXatLxBI9gBaebbnrfifHhDYfgasaacH8akY=wiFfYdH8Gipec8Eeeu0xXdbba9frFj0=OqFfea0dXdd9vqai=hGuQ8kuc9pgc9s8qqaq=dirpe0xb9q8qiLsFr0=vr0=vr0dc8meaabaqaciaacaGaaeqabaqabeGadaaakeaadaqdaaqaaiabd2eanjabd2eanbaaaaa@2F03@ > *PM*_B_, *PM *fluorescence values for that allele are set to 0. In such instances ratios produce monomorphic RAS scores. If MM¯
 MathType@MTEF@5@5@+=feaafiart1ev1aaatCvAUfKttLearuWrP9MDH5MBPbIqV92AaeXatLxBI9gBaebbnrfifHhDYfgasaacH8akY=wiFfYdH8Gipec8Eeeu0xXdbba9frFj0=OqFfea0dXdd9vqai=hGuQ8kuc9pgc9s8qqaq=dirpe0xb9q8qiLsFr0=vr0=vr0dc8meaabaqaciaacaGaaeqabaqabeGadaaakeaadaqdaaqaaiabd2eanjabd2eanbaaaaa@2F03@ > *PM*_A _AND MM¯
 MathType@MTEF@5@5@+=feaafiart1ev1aaatCvAUfKttLearuWrP9MDH5MBPbIqV92AaeXatLxBI9gBaebbnrfifHhDYfgasaacH8akY=wiFfYdH8Gipec8Eeeu0xXdbba9frFj0=OqFfea0dXdd9vqai=hGuQ8kuc9pgc9s8qqaq=dirpe0xb9q8qiLsFr0=vr0=vr0dc8meaabaqaciaacaGaaeqabaqabeGadaaakeaadaqdaaqaaiabd2eanjabd2eanbaaaaa@2F03@ > *PM*_B_, no interpretable signal is obtained (because the denominator is 0). In such instances the quartet was not used in subsequent analyses. Only SNPs that retained at least 70% of their available quartets were used in subsequent analyses (i.e., 5/6 or 7/10 quartets). Approximately 96% of SNPs are retained using this criterion, depending on the successfulness of the assay.

Allele frequency estimates for the 500 K microarray set were calculated manually from the raw probe intensity data exported as a .txt file, for reasons outlined previously [33]. In this study, however, we used a modified version of the RAS score algorithm that is based on an average of all quartet measures (RAS_av-all_) rather than deriving separate RAS scores for sense and anti-sense quartets. The rationale for using RAS_av-all _was twofold: Sense and anti-sense measures should not differ systematically and a composite measure should be more reliable.

### Analysis

Reliability was assessed in relation to the average correlation between the 5 DNA pools across all SNPs. Validity was assessed by comparing average allele frequency estimates across 5 DNA pools to those from the independent sample available from HapMap [11] and NetAffx™ [48]. The sample included 60 unrelated individuals from CEPH trios (30 mothers and 30 fathers) who were genotyped using the Affymetrix 500 K GeneChip^® ^for the HapMap project [11].

### Artificially constructed pooling experiment

To evaluate the level at which the observed SNP-MaP inter-chip reliability might compare to an ideal individual genotyping scenario, a simulated pooling experiment involving unrelated individuals was conducted using the genotypes of CEPH parents deposited in HapMap. Two independent pools were constructed: one comprising 30 CEPH mothers, the other 30 CEPH fathers. The allele frequencies of the pools were calculated separately and then correlated with each other using SPSS.

## Authors' contributions

SD wrote the manuscript and co-analysed the data. LB ran the microarrays, co-wrote the manuscript and co-analysed the data. LS co-wrote the manuscript and co-analysed the data. RP is principal investigator, conceived and designed the study and co-wrote the manuscript. All authors read and approved the final manuscript.
